# Identification and Characterisation of Simiate, a Novel Protein Linked to the Fragile X Syndrome

**DOI:** 10.1371/journal.pone.0083007

**Published:** 2013-12-11

**Authors:** Kristin Derlig, Andreas Gießl, Johann Helmut Brandstätter, Ralf Enz, Regina Dahlhaus

**Affiliations:** 1 Institute for Biochemistry, Emil-Fischer Centre, University of Erlangen-Nuremberg, Erlangen, Germany; 2 Department of Biology, Animal Physiology, University of Erlangen- Nuremberg, Erlangen, Germany; Hokkaido University, Japan

## Abstract

A strict regulation of protein expression during developmental stages and in response to environmental signals is essential to every cell and organism. Recent research has shown that the mammalian brain is particularly sensitive to alterations in expression patterns of specific proteins and cognitive deficits as well as autistic behaviours have been linked to dysregulated protein expression. An intellectual disability characterised by changes in the expression of a variety of proteins is the fragile X syndrome. Due to the loss of a single mRNA binding protein, the Fragile X Mental Retardation Protein FMRP, vast misregulation of the mRNA metabolism is taking place in the disease. Here, we present the identification and characterisation of a novel protein named Simiate, whose mRNA contains several FMRP recognition motifs and associates with FMRP upon co-precipitation. Sequence analysis revealed that the protein evolved app. 1.7 billion years ago when eukaryotes developed. Applying antibodies generated against Simiate, the protein is detected in a variety of tissues, including the mammalian brain. On the subcellular level, Simiate localises to somata and nuclear speckles. We show that Simiate and nuclear speckles experience specific alterations in FMR1^-/-^ mice. An antibody-based block of endogenous Simiate revealed that the protein is essential for cell survival. These findings suggest not only an important role for Simiate in gene transcription and/or RNA splicing, but also provide evidence for a function of nuclear speckles in the fragile X syndrome. Indeed, transcription and splicing are two fundamental mechanisms to control protein expression, that underlie not only synaptic plasticity and memory formation, but are also affected in several diseases associated with mental disabilities.

## Introduction

For all living cells and every organism, it is vital to strictly regulate protein expression in response to environmental cues and signals as well as to control its own development, for example during mitosis and differentiation or during synapse formation in the maturing brain. Hence, evolution has brought about a multitude of molecular mechanisms ranging from DNA organisation to post-translational protein modifications, which ultimately serve to precisely regulate the expression of each protein encoded by the app. 20,000 genes present in any eukaryotic cell at any time. Indeed, more than 40 years ago it has already been suggested that the phenotypic diversity observed within and between species is brought about by differences in protein expression patterns rather than structural differences in genes [1-3].

Given the sensitivity and complexity of the mammalian brain it is therefore not surprising that intellectual disabilities have been associated with altered protein expression patterns and deficits in the mRNA metabolism. For instance, chromosomal rearrangements involving VCXA (variable charge x linked protein A), a protein modulating mRNA decapping and thus, mRNA stability and translation [4], have been connected with cognitive deficits (CDs), whereas point mutations affecting the protein were not found in patients with CDs yet [5,6], implying that alterations in the expression of VCXA are causing the impairments. Also, a single-copy chromosomal deletion of a region encoding Neurexins, presynaptic cell-adhesion molecules, that function in synapse formation and maturation (reviewed in 7), has been associated with autism [8]. In addition, autistic patients have been found to often carry novel gene duplications and deletions in their genomes [9], suggesting that the two, enhanced and diminished expression of specific proteins can conduce to the manifestation of autism. 

A disease characterised by an extensive misregulation of protein expression is the fragile X syndrome (FXS; reviewed in 10,11). Though designated by both, cognitive deficits and autistic behaviours, FXS is caused by the loss of a single protein only, the Fragile X Mental Retardation Protein FMRP (reviewed in 12,13). Holding three RNA-binding domains, FMRP is able to interact with a variety of mRNAs by binding to several different RNA-motifs including U-pentamers [14], the Kissing complex [15], SoSLIP [16], and G-rich regions/G-quadruplexes [17-20]. Indeed, it has been estimated that thereby, the expression of 4% of all proteins in the mammalian brain is influenced by FMRP [21], however, the relevance of most proteins dysregulated in FXS for the course of the disease has not been investigated yet. 

The functions of FMRP in regulating protein expression are far-reaching: FMRP modulates the synthesis of novel proteins at several different stages of the mRNA metabolism lasting from early stages of DNA transcription in the nucleus to mRNA translation at synapses (for a comprehensive review, please see 22). For instance, FMRP can directly repress or enhance the translation of its partner mRNAs, yet as well it is involved in retro- and anterograde transport of mRNA particles between the nucleus and synaptic sites. Furthermore, FMRP has recently been shown to not only regulate chromatin structures, but to also associate with active transcription sites, where it binds to nascent mRNA [12,23,24] and may even take action in alternative splicing since G-quadruplexes present in the mRNA of FMRP itself were found to function as exonic splicing enhancers [25].

The molecular machinery required for both processes, DNA transcription and RNA splicing, is organized in specific compartments, named nuclear speckles [26]. Correspondingly, nuclear speckles are often observed in association with active transcription sites [27-29] and it has hence been proposed that nuclear speckles act as organisation centres which orchestrate active genes at their periphery [27]. However, FMRP was not found in nuclear speckles.

Nuclear speckles are highly dynamic protein ensembles that quickly alter their size, shape and number in response to changes in gene expression and RNA splicing. Inhibition of transcription, for instance, leads to an increase in speckle size [30,31], whereas enhanced transcription, such as observed during viral infection or expression of intron rich genes [32-35], results in a redistribution of transcription and splicing factors to active genes and thus, a decrease in speckle size, but an increase in speckle numbers. Photobleaching experiments revealed that nuclear speckles experience a rapid turnover rate with a complete signal recovery after 30-50 seconds depending on the specific protein studied [36,37]. Taken together, these results suggest a model where transcription - as well as splicing factors constantly ply between active genes and the interior of nuclear speckles or become engaged in the formation of new speckles, while the general speckle structure is built up by protein-protein interactions regulating the association and dissociation rates. 

DNA transcription and RNA splicing are two processes highly relevant to synaptic plasticity and FXS, since specific forms of long-term potentiation (LTP) and long-term depression (LTD) have been shown to be altered in FXS (for a review please see 38), and both forms of plasticity ultimately rely on protein synthesis and gene transcription. However, despite some alterations in the mGluR-dependent MAPK (Mitogen-activated protein kinase) signalling pathway [39] and the mTOR-signalling cascade [40] the mechanisms by which FMRP may regulate DNA transcription and RNA splicing have remained elusive. Given the far-reaching functions of FMRP in the mRNA metabolism and the relevance of DNA transcription and RNA splicing to protein expression and long term synaptic plasticity, we therefore speculated that there will be further signalling mechanisms to control and coordinate gene expression in a FMRP dependent manner, for example during late phase LTP or mGluR-mediated LTD. To address this issue, we searched for proteins regulated by FMRP and likely to have a nuclear function. We found a thus far unknown protein of 22kDa, which we named Simiate.

Here, we now report a characterisation of the protein. Simiate most likely arose app. 1.7-1.85 billion years ago when eukaryotes evolved and localises to somata as well as to nuclear speckles. Using our newly generated Simiate antibodies to perform 3D reconstructions of Simiate in wildtype and FMR1^-/-^ mice, a model of FXS, we demonstrate brain region specific changes in nuclear speckles of FMR1^-/-^ animals. An antibody based block of endogenous Simiate revealed that the protein is not only vital for cells, but also suggests that Simiate functions as a transcription and/or splicing enhancer, thus implying Simiate to be involved in gene expression regulation. 

## Methods

### 2.1: Molecular biology and protein biochemistry

#### 2.1.1: Generation of Simiate constructs

Using specific primers (5’-ggaattcATGGAAGAGCTCCGCTGC and 5’-acgcgtcgacTCAGGGCGTGGTGGCTG), Simiate was cloned from homemade mouse brain cDNA into pGEX-4T-1 (*GE Healthcare*) and pMAL-c2g (*New England Biolabs Inc.*) -vectors for bacterial expression as well as into pEGFP (*Clontech*) and pCMV5-FLAG (generous gift from Prof. M. Wegner) vectors for expression in mammalian cells. 

#### 2.1.2: Antibodies

***Primaryantibodies*** *:* Calnexin (rabbit, *Abcam*; WB 1:2000), FLAG (mouse, *Sigma*; IHC 1:100), FMRP (goat*, Abcam*; IHC 1:200), GFP (mouse; *Covance*; WB 1:2000), Gephyrin (generous gift from Dr. Volker Eulenburg; IHC 1:400), MAP2 (chicken, *Abcam*; IHC: 1:2500), Simiate (rabbit, *homemade*; WB: 1:2000; IHC: 1:200, guinea-pig, homemade, WB: 1:2000; IHC: 1:200), NeuN (mouse, *Abcam*; IHC 1:500), SAP97 (rabbit, *ABR*; 1:200), SC35 (mouse, *Santa Cruz*; IHC: 1:250)

***Secondaryantibodies*** *:* HRP antibodies (*GE Healthcare*; WB 1:2000), Alexa-antibodies (*Life technologies*; IHC 1:500-1:1000), gtαmCy5 (*Abcam*; IHC 1:250)

#### 2.1.3: Fusionprotein production

Glutathione-S-Transferase (GST)- as well as maltose-binding-protein (MBP)- Simiate constructs were expressed in *E. coli* BL21 Rosetta (*Novagen*). Following induction with 0.5mM isopropyl-beta-D-thiogalactoside (IPTG, *Sigma*) for GST fusion proteins or 0.3mM IPTG and 0.2% glucose for MBP fusion proteins, cells were lysed in a french press (*Thermo Electron*) and recombinant proteins were purified according to the manufacturer's instructions (GST: *GE Healthcare*, MBP: *New England Biolabs*). Applying SDS-PAGE in combination with a BSA standard and Coomassie Brilliant Blue R-250 (*Serva*) staining, the quality and quantity of all obtained fusion proteins were determined. 

#### 2.1.4: Antibody purification

In order to detect endogenous Simiate, antibodies against GST-Simiate were raised in rabbit and guinea pig (*Pineda*) and subsequently affinity-purified from sera using CNBr Sepharose fast flow beads (*GE Healthcare*) carrying covalently coupled MBP-Simiate. After three alternate washings with 0.1M sodium acetate (pH 4.0) and 0.1M tris/0.5M NaCl (pH 8.0) antibodies specific to Simiate were eluted using 0.1M glycine (pH 2.6) and immediately neutralized with 6M tris (pH 8.0). The obtained antibody solution was stored with 50% glycerol at -20 °C until usage. 

#### 2.1.5: Immunoprecipitations

To examine the expression of Simiate, the protein was precipitated from diverse mouse tissue samples using the newly generated polyclonal antibody. Pre-immune serum served as control. The IgGs were covalently coupled to protein A agarose (*Roche Applied Science*) using the following procedure: incubation of 50µl agarose A and 20 µl rbαSimiate antibody or 20µl pre-immune serum in PBS-T (1% Tween-20 in Phosphate buffered Saline (PBS)) for 3h at 4°C; wash 2x with PBS-T, wash 2x with triethanolamine (TEA, 0.2M pH 8.2), incubation in TEA with additional 20mM diemethyl-pimelimidat-HCl (pH 8.2) for 45min at 20°C, wash 1x with TEA, incubation with 20mM ethanolamine, pH 8.2, for 10 min at 20°C and wash 1x with Hepes-buffer (10mM HEPES, pH 7.5; 1mM EGTA; 0.1mM MgCl_2_; 1% triton; 150mM NaCl) with protease inhibitor (complete ULTRA Tablets, EDTA-free, *Roche Applied Science*). All spins during the procedure were carried out at 500g and 4°C for 1min. Cell lysates from various tissues were prepared as published previously [41] and kept with the coated matrix overnight at 4°C end over end. Hereafter, the samples were washed 3x with Hepes-buffer before all retained proteins were eluted for 10min at 60°C with 4xSDS buffer (20% SDS, 40% glycerol, 250mM TRIS, pH 6.8) and analysed using specific antibodies to detect proteins of interest by western blotting. In order to also rate the expression level of Simiate in all analysed tissues, the total protein content of each lysate was measured as integrated density on a Coomassie stained acryl amide gel including proteins from app. 10-200kDa and normalised to the tissue with the highest protein content (liver).

#### 2.1.6: Co-immunoprecipitations with RNA

For a detailed description of the coprecipitation assay with FMRP and mRNA please refer to [42]. In brief, endogenous FMRP was precipitated from mouse brain lysate and incubated with prepared mRNA over night. Following three washing steps and elution, the presence of Simiate-mRNA was proven by RT-PCR using RNA as well as water only as negative controls and the following primer pair: 5'-atggaagagctccgc and 5'-tcagggcgtggtggc. For ARMC1, the primer pair 5'-atgaattcatcctcttctact and 5'-tattgctgacgccagagcct was used.

### 2.2: Histochemistry

#### 2.2.1: Culture and immunohistochemistry of human embryonic kidney (HEK-293) cells

HEK-293 cells were cultured in Minimum Essential Media (*Life technologies*; containing 10% fetal calf serum) at 37°C and 5% CO_2_. 

To test the sensitivity and specificity of the rbαSimiate antibody, we expressed GFP-Simiate as well as GFP in HEK-293 cells using JetPei (*Polyplus transfection*) for transfection as recommended by the producer. HEK-293-lysates were prepared as published previously [43], subjected to SDS-PAGE and analysed via western blotting. Following Coomassie staining, the concentration of recombinant proteins was calculated according to a BSA standard.

For immunofluorescence experiments, the cells were seeded on poly-L-lysine-coated glass coverslips, grown for 24 hours and transfected as stated above. One day after transfection, the cells were washed gently with PBS (containing 0.5mM MgCl_2_ and 0.5mM CaCl_2_ and preheated to 37°C), fixated for 10min using 4% paraformaldehyde (PFA, 37°C) and quenched for 30min by applying PBS containing 0.5mM MgCl_2_, 0.5mM CaCl_2_ and 25mM glycine. After an additional washing step with PBS, the coverslips were placed in blocking solution (10% fetal calf serum, 2% BSA, 0.2mg/mL Saponin in PBS) for 1h, whereupon the primary and secondary antibodies were applied in the same manner, but with 3 washing steps in between. Finally, all coverslips were mounted on object plates using Aqua Poly Mount (*Polysciences*) and imaged either on a non-confocal Axiophot or a confocal microscope (Laser Scanning Microscope T-PMT, both *Zeiss*).

#### 2.2.2: Primary culture and immunofluorescence of neurons

Dissociated neuronal cultures were prepared from hippocampi of E17-E18 rat brains as described in [42] for newborn mice and seeded on poly-L-lysine–coated glass coverslips (*Menzel Gläser*) in required densities. The Neurobasal medium (*Life technologies*; containing 1xB27, 5mM L-glutamine and 1xPenicilline/Streptomycine) was replaced half two times a week. All immunofluorescence stainings were performed as specified for HEK-293 cells in section 2.21. 

#### 2.2.3: Immunohistochemistry of mouse brain slices

In order to perform immunohistochemistry experiments with FMR1^-/-^ as well as wildtype mice, freshly harvested mouse brains were processed and stained as published in [42]. Alexa-antibodies alone served as negative control.

#### 2.2.4: Blockage of Simiate

To address the function of Simiate in vivo, we blocked the endogenous protein in HEK-293 cells by applying chariot reagent (*Active Motif*) as advised by the manufacturer to shuttle the rbαSimiate antibody into the cells. In brief, the appropriate amount of rbαSimiate was preincubated 30min at RT with rbαAlexa568 (a corresponding amount of rbαAlexa568 alone served as negative control) and incubated for another 30min with chariot. After washing the cells with PBS, the antibody-chariot-complexes were diluted in an equal volume of MEM (37°C, without additives) and applied to the cells, allowing them to grow for 3h more while after 1h, 10% fetal calf serum were added. Immunohistochemistry experiments were implemented as detailed under 2.31. To quantify the amount of unblocked Simiate epitopes in HEK-293 we measured the Simiate signal as integrated density of the corresponding immunofluorescent signal using ImageJ. The Simiate epitopes in rbαSimiate treated cells were determined by measuring the total Simiate signal and subtracting the signal of antibody-chariot-complexes. By comparing the determined integrated densities of treated cells to control treated cells we calculated the Simiate epitopes not targeted by rbαSimiate antibodies. In order to identify apoptotic cells, a TUNEL staining (*Roche Applied Science*) was employed as suggested by *Roche Applied Science.*

### 2.3: Statistics

All statistical calculations were carried out in Prism (*GraphPad Software Inc.*) and Excel (*Microsoft Corp.*). The significance levels of each test are reported as significant for p<0.05 (*), as highly significant for p<0.01 (**) and as extremely significant for p<0.001 (***). 

The homoscedasticity of two groups was analysed by performing f-testing. Following an insignificant outcome, a potential difference in the means of the two groups was analysed by a two-tailed Student's t-test (t-test). For comparing more than two groups, one-way ANOVA was applied. In this case, the origin of an observed significance was further evaluated by implementing Bonferroni post-hoc testing. This data is represented as means with 95% confidence intervals in all corresponding figures. 

Given the significant inhomoscedasticity observed in the 3D data set (f-test p<0.05), the non-parametric Kruskal-Wallis test (H) was chosen to compare medians. Post-hoc testing was performed by Dunn's multiple comparison test. Accordingly, this data set is shown as median with 10 and 90% quantils. For the comparison of FXS and wildtype data, the non-parametric Mann-Whitney-u test (u-test) was employed.

Categorical data such as the Simiate localisation was analysed by contingency tables and Fisher's exact test for 2x2 tables or else, the Chi^2^ test. Here, the 95% confidence interval was calculated according to [44].

### 2.4: Bioinformatics

Potential G-quadruplexes present in the mRNA of Simiate were identified using the web tool Quadruplex forming G-Rich Sequences mapper, while motifs located in the protein itself were predicted by applying The Eukaryotic Linear Motif resource for Functional Sites in Proteins with standard settings.

### 2.5: Animal Care and Ethics Statement

All mice were kept at the rodent facility of the Institute for Biochemistry in strict accordance with the guidelines of the Federation of European Laboratory Animal Science Associations and the recommendations of the European Convention for the Protection of Vertebrate Animals used for Experimental and Other Scientific Purposes of the Council of Europe. Protocols were approved by the Animal Care Committee of the University of Erlangen-Nuremberg (Permit number: TS-8/12 Biochemie) and animal welfare conventions are controlled regularly by the city of Erlangen. Breeding of FMR1^-/-^ (C57BL/6) and wildtype (C57BL/6) mice was implemented in line with the recommendations of Berry & Linder ([45]; cp. also [42]). The mice were housed under a 12 hour light-dark cycle with constant temperature in groups of 2 to 5 individuals and with standardised enrichment including nesting material and pipes to allow for species specific behaviour. All experiments included only adult animals, 2.5 to 6 months of age, sex- and age matched, and were analysed genotype-blinded. Wildtype mice used for breeding were obtained from Charles River Laboratories.

## Results

### 3.1: Simiate

By screening data bases for mRNAs containing G-quadruplexes suitable to be recognized by FMRP, we recently identified several potential FMRP target mRNAs [42,46], among others a thus far unknown protein, which we named Simiate (constringed from *simia*, Latin, a female ape, and M, A, T and E, the first four amino acids of the ape orthologs). The mRNA of Simiate displays 11 potential G-quadruplexes that may mediate an association with FMRP [46-48] and which are localised in the 5' (4 G-quadruplexes) and 3' (7 G-quadruplexes) untranslated region (UTR) of the transcript. In addition, 8 U-pentamers, another structure known to be recognized by FMRP (reviewed in 49), are also included in the 5' and 3' UTR of the Simiate-transcript (1 and 7, respectively). 

**Figure 1:**
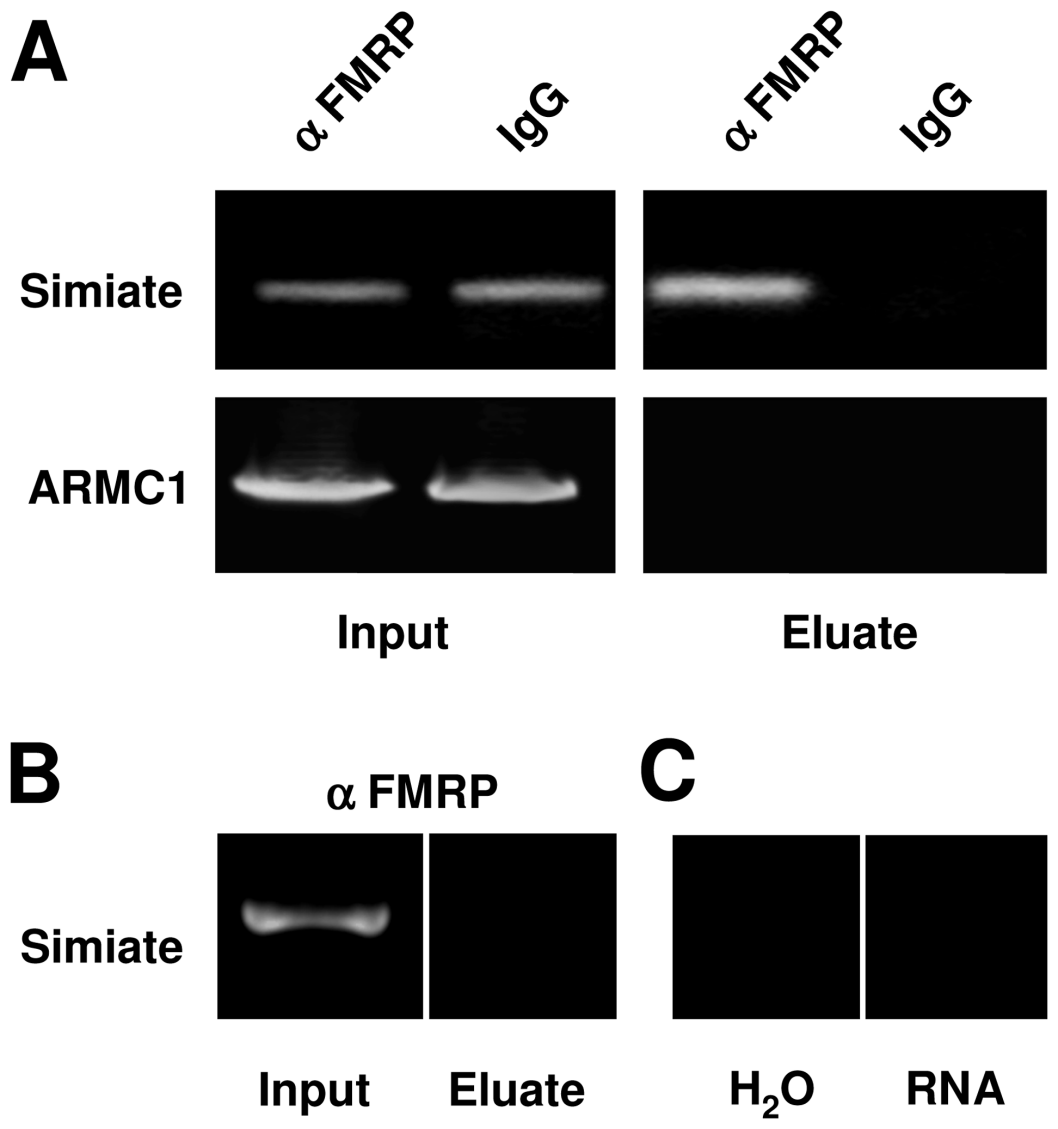
Simiate. A) Co-immunoprecipitation of FMRP and Simiate- or ARMC1-mRNA visualized by RT-PCR. IgG serves as negative control for the assay. B) The same experiment as shown in A, but the immunoprecipitation of FMRP is implemented with FMR1^-/-^ mice. C) Negative controls for the reaction showing that the association requires not only FMRP (cp. panel B), but also transcribed Simiate-mRNA.

In order to verify the implied interaction of FMRP and the mRNA of Simiate, we performed co-immunoprecipitations of endogenous FMRP precipitated from mouse brain cytosol by FMRP-specific antibodies and purified mRNA, while IgG, which does not precipitate FMRP, served as negative control (Figure 1, also see 42). Using RT-PCR, we could demonstrate a specific copurification of FMRP and Simiate-mRNA (Figure 1A, upper panel), whereas the unrelated ARMC1-mRNA (Armadillo-repeat containing protein1) did not display an association with FMRP under the employed conditions (Figure 1A, lower panel), hence confirming the specificity of the experiment. An immunoprecipitation from FMR1^-/-^ mice (Figure 1B) illustrates the requirement of FMRP and mRNA (Figure 1C) for the association. In parallel, an interaction of FMRP with Neuroligin2-mRNA was also shown (published previously in [42]). Since this interaction is reaffirmed by the data of Darnell et al. [4], Ascano et al. [17] and Liao et al. [50], the outcome further supports our finding.

Given the unknown nature of Simiate and its potential relevance to FXS, we decided to investigate the protein in more detail. Simiate is a rather small protein with a calculated molecular weight of 21.6 kDa, which nonetheless contains several predicted consensus sequences for protein-protein interactions, e.g. for associations with SH2/3 or FHA1/2 domain (Src homology or forkhead-associated, respectively) comprising molecules. 

Next, we assessed the prevalence of Simiate in different species by screening genomic data bases (Figure S1). It turned out that Simiate is present in manifold groups including mammals, birds, reptiles, fish, insects, divers invertebrates and even plants and protists. Some groups show characteristic amino acid cassettes at the N-terminus of Simiate (e.g. plants and insects, but also rodents), while the C-terminal part is more conserved. Here, a cluster of hydrophobic and acidic amino acids preceded by a small amino acid (aa 131–140 of the murine isoform) is distinctive for all Simiate orthologs. 

**Figure 2:**
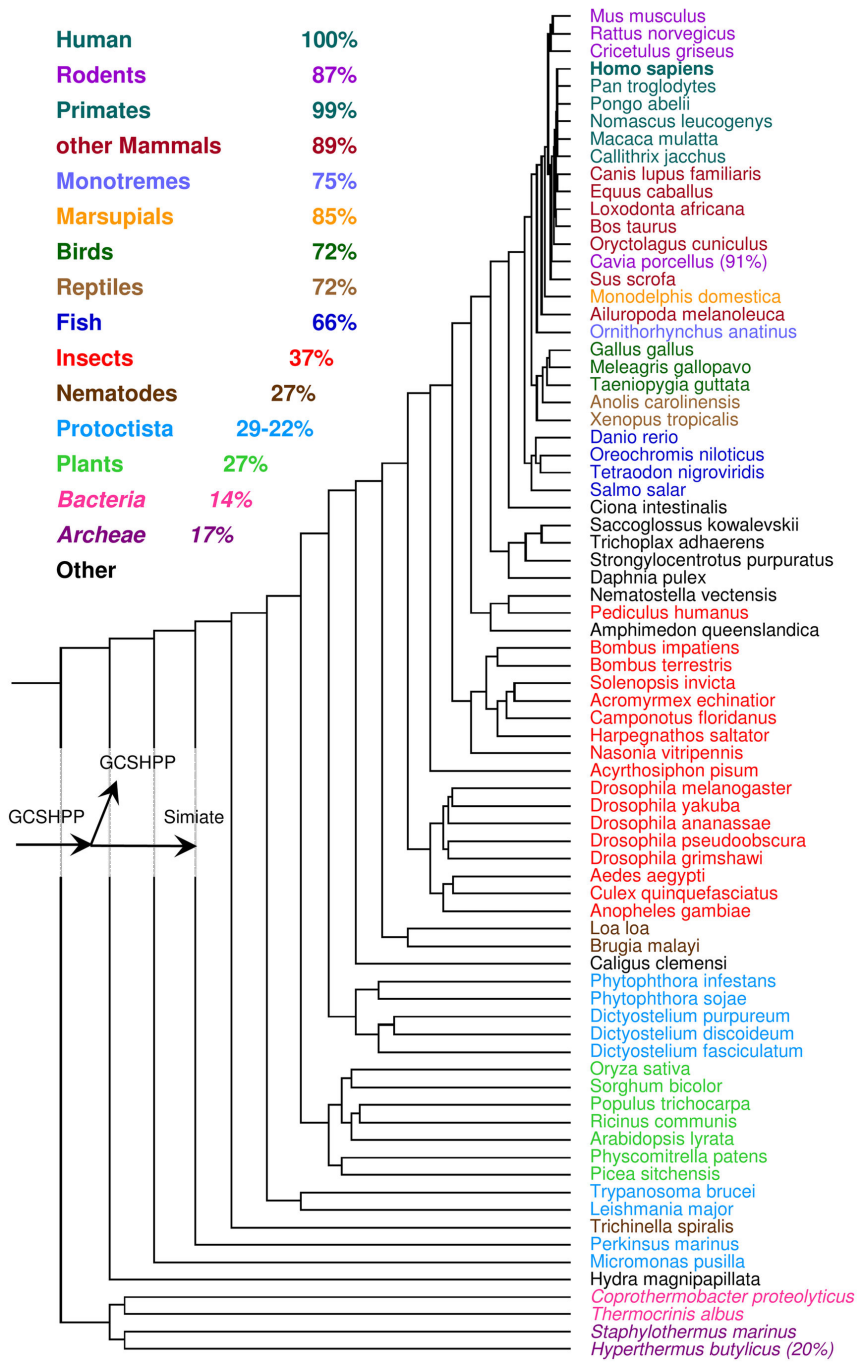
Simiate orthologs. A phylogenetic tree of Simiate. The averaged percentage of identities shared with the human ortholog (=100%) is shown for diverse groups. Please note that bacteria and archaea express GCSHPP (glycine cleavage system H protein, mitochondrial precursor) only, therefore the corresponding numbers refer to the alignment with GCSHPP and are presented in italic letters. Species demonstrating exceptional identity percentages are shown along with the actual percentage in brackets. All orthologs have been identified using the murine isoform as a template. Murine isoform: cDNA sequence BC026590, location: C4B3; 4, GI:124487245. Human isoform: gene *FAM206A*, location: C9orf6, GI:205277341; current data base status: predicted protein, evidence at transcript level).

Though Simiate is present in a wide variety of species, it is absent from bacteria and archaea. Instead, these species carry a protein named mitochondrial precursor of glycine cleavage system H protein (GCSHPP, Figure S1 and Figure 2: italic letters), which shares about 20% identity and 40% similarity with the protistian Simiate and is still present in mammals, suggesting that this protein is the ancestor of Simiate. Given that bacteria and archaea already lived on our planet for app. 1.65 billion years when about 1.7 - 1.85 billion years ago first protists occurred [51-53] and given the presence of Simiate in analysed eukaryotic organisms only, it is likely that Simiate arose during the evolution of eukaryotes from prokaryotes. 

Interestingly, further sequence analysis revealed that FMRP is also an eukaryotic protein, though somewhat younger than Simiate. While bacteria and archaea miss FMRP as well, the protein is present in eukaryotic cells such as the protists *Salpingoeca* sp. and *Monosiga brevicollis*, which are close relatives of the first multicellular animals, or in the green alga *Micromonas pusilla* and the red alga *Guillardia theta*. By contrast, the red alga *Hemiselmis andersenii* as well as the green algae *Chlamydomonas reinhardtii* and *Mesostigma viride* lack FMRP, as do the descendents of *Mesostigma*; land plants. These findings imply that FMRP arose after the occurrence of first eukaryotes and near to the development of multicellular animals, however, since the G-quadruplex present in the 3’ UTR of the FMRP transcript itself first occurred in modern mammalia (*Eutheria*), it is likely that parts of the mechanisms regulating the expression of FMRP evolved more recently and are less ubiquitous.

The rise of eukaryotes from prokaryotes represents an important step in evolution since these cells introduced a more complex organisation including the invention of mitochondria, chloroplasts and a well-organized genome in the nucleus. Given the small size of Simiate and the fact that proteins of less than app. 40kDa freely enter the nucleus via diffusion [54], these findings suggest that Simiate functions in nuclear organisation.

### 3.2: Simiate expression

In order to study Simiate more meticulously, we generated Simiate immune sera in rabbit (rb) and guinea pig (gp) and affinity purified antibodies against Simiate. The specificity of our polyclonal gpαSimiate as well as rbαSimiate antibodies were then assessed in immunofluorescence and western blotting experiments (Figure S2A-E: rb and S2A'-E': gp). Therefore, we expressed GFP-Simiate (Figure S2A, A') in HEK-293 cells, preserved the cells with PFA, and stained them with our rb- as well as gpαSimiate antibodies. As it can be seen from the magnifications, both antibodies specifically recognize GFP-Simiate, whereas GFP solo is not detected (data not shown). Also, preincubation of both antibodies with GST-Simiate abolishes the signal (Figure S2B, B') completely. Similar results are obtained by western blotting (Figure S2C, C'): While GFP-Simiate is specifically detected by our two antibodies, neither GFP nor any other HEK-293 cell proteins are recognized. Again, the signal can be blocked entirely by preincubation of rb- and gpαSimiate with GST-Simiate. Western blots with endogenous proteins further confirm the specificity of both antibodies, here, they detect a single band corresponding to the molecular weight of Simiate in whole brain cytosol (Figure S2D, D'). In order to estimate the detection level of rb- and gpαSimiate, we now offered different amounts of GFP-Simiate to the antibodies on a western blot (Figure S2E, E'). This experiment revealed that the minimum amount of Simiate recognized is about 20ng in both cases. Taken together, the data illustrates that endogenous and recombinant Simiate are specifically detected in their native and denatured forms by both antibodies, thereby providing two independent proofs for the expression of Simiate in brain.

**Figure 3:**
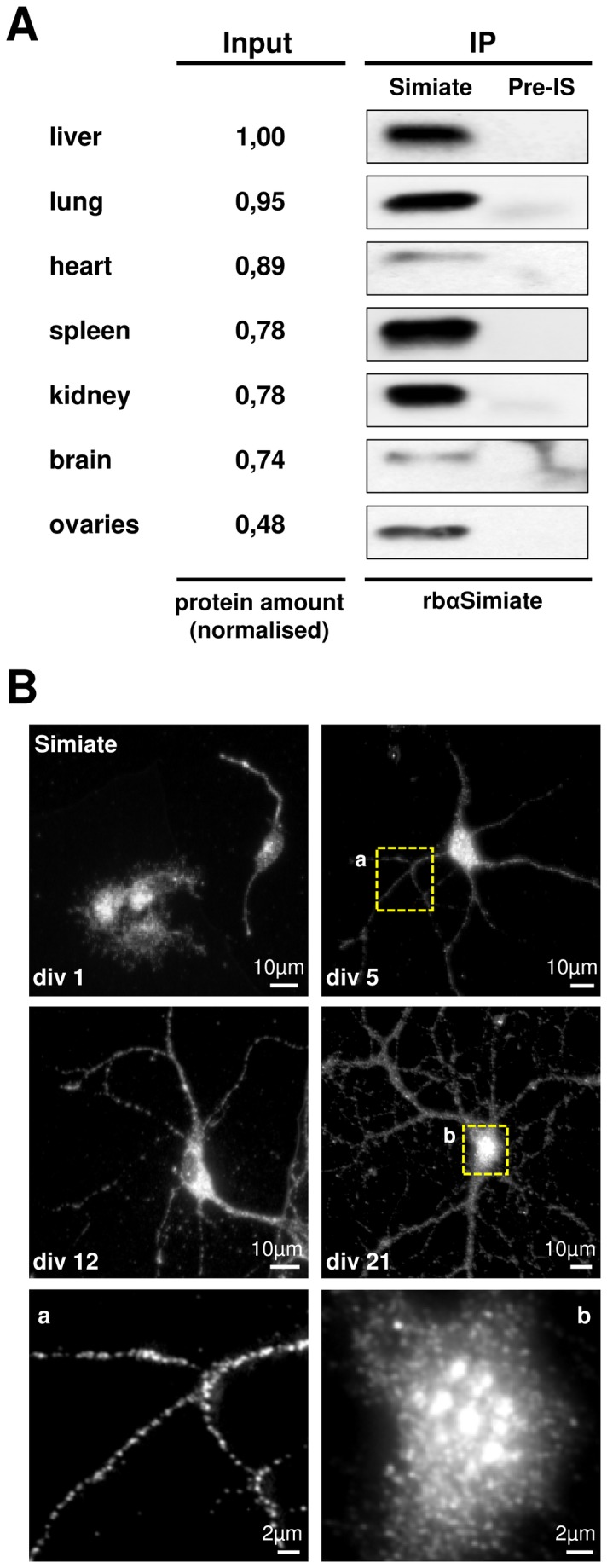
The expression of Simiate. A) Simiate is present in several different tissues. Endogenous Simiate was immunoprecipitated from different mouse organs using rbαSimiate and subjected to western blotting. Pre-immune serum (Pre-IS) served as negative control. Differences in the amount of protein available to immunoprecipitation (“input”) are displayed as total quantities of proteins normalised to the sample containing the highest quantum of protein (liver). B) Representative immunofluorescence pictures demonstrating the expression of endogenous Simiate during the development of primary hippocampal neurons. The numbers indicate the corresponding day in vitro (div). At the bottom, magnifications of a dendrite (a) and a nucleus (b) are shown.

We next asked in which tissues Simiate is present. Since the amount of total protein extracted is very low in some tissues (e.g. ovaries), Simiate was first enriched by IP. Therefore, we covalently coupled the rbαSimiate antibody to protein A-Agarose and precipitated Simiate from lysates of numerous mouse organs. In order to also rate the amount of Simiate available in each tissue, we compared the quantity of precipitated Simiate to the amount of total protein available in each sample (input) on a western blot (Figure 3A). This experiment revealed that Simiate is expressed in all analysed tissues, but to a different extend. Although only a relative small amount of protein is available in ovaries, Simiate is distinctly detected in this tissue, suggesting a rather high expression of Simiate in ovaries. On the other hand, in heart and brain only little Simiate is seen despite the adequate amounts of protein observed, implying a low expression level. Average expression levels are seen in spleen, kidney, liver and lung. The ubiquitous presence of Simiate in all analysed tissues is in line with our *in silico* analysis illustrating that Simiate is a protein distinctive for all eukaryotic cells.

Furthermore, we investigated the onset of Simiate expression during development using primary hippocampal cultures (Figure 3B). The experiments show that Simiate is present in neurons and glia ever since the first day of cultivation. Simiate localises to the nuclei and somata of both cell types and occasionally clusters inside the nucleus. Following day in vitro (div) 5, small Simiate clusters can also be observed in dendrites, however, in div 21 neurons, no colocalisation with SAP97 or Gephyrin is seen (data not shown), confirming that Simiate is neither localised to excitatory nor inhibitory synapses. 

### 3.3: Simiate and FXS

**Figure 4:**
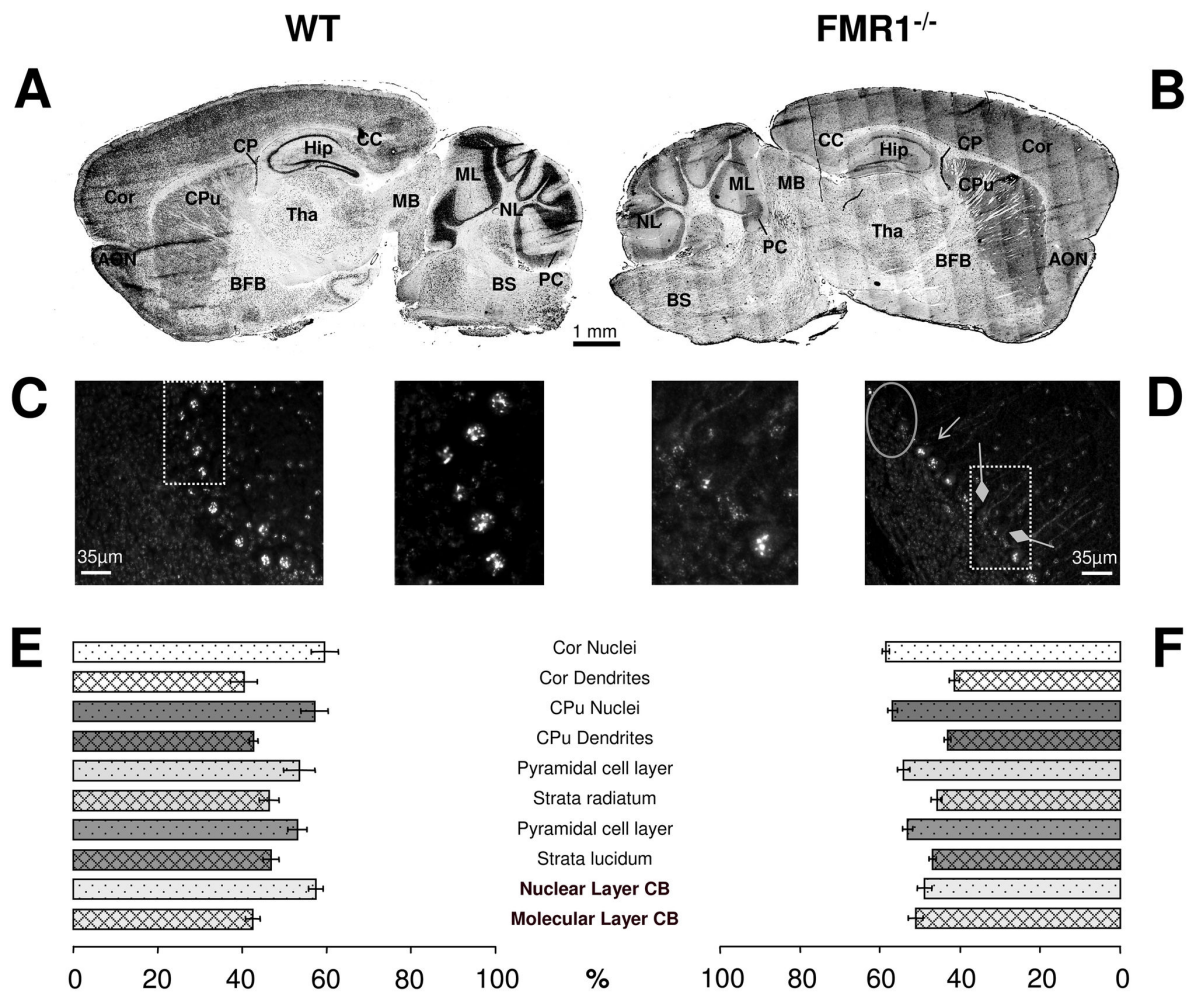
Simiate in the mammalian brain. A) An immunofluorescence picture illustrating the expression of Simiate in the adult murine brain. The picture has been reconstructed from a number of 10x microscopic photographs and is shown colour inverted. B) The expression of Simiate in FMR1^-/-^ mice. C,D) Purkinje cell layer of the Cerebellum in wildtype (C) and FMR1^-/-^ (D) mice. The circle outlines an area lacking Purkinje cells, while the rhombic tipped arrows indicate cells with distinctly reduced Simiate clustering in the nucleus. E,F) Quantification of protein levels in different brain regions of wildtype (E) and FMR1^-/-^ animals (F). The bars display the signal allocation between nuclei and neuropil of each brain region analysed in percent. Statistical significance was tested using a two-tailed t-test to compare FMR1^-/-^ and wildtype mice (n=8 slices from 3 mice each (N=3 for wildtype and FMR1^-/-^ animals)). Brain regions with significant differences between wildtype and FMR1^-/-^ mice are shown in bold letters (p<0.001). AON: anterior olfactory nucleus, BFB: basal forebrain, BS: brain stem, CPu: Caudoputamen, CP: Cori plexus, CC: Corpus callosum, Cor: Cortex, Hip: Hippocampus, MB: midbrain, ML: molecular layer of the Cerebellum, NL: nuclear layer of the Cerebellum, PC: Purkinje cell, SPF: striatopallidal fibres, Tha: Thalamus, wt: wildtype.

Given that the mRNA of Simiate is recognized by FMRP (see Figure 1A) and that FMRP functions in the transport and translation of its partner mRNAs, we hypothesized that the loss of FMRP in FXS may influence the expression of Simiate. Therefore, we first examined the expression and localisation of Simiate in the brain of wild-type mice in more detail (Figure 4). While present in all brain regions (Figure 4A), Simiate is most abundant in the Hippocampus, the nuclear layer of the Cerebellum, the Cortex and the Caudoputamen. Lowest levels of Simiate expression are observed in the brainstem, the midbrain and the basal forebrain. Next, we performed immunofluorescence stainings of FMR1^-/-^ mouse brains (Figure 4B). Although no major abnormalities in the expression pattern are observed, a striking loss of immunofluorescence intensity throughout the brain is evident. This result may reflect a reduction in Simiate expression. Indeed, a western blot analysis of brain homogenates, each pooling six individuals, further supports the finding (Figure S3). 

A more detailed look revealed that Simiate is localised in nuclei and dendrites throughout the brain. It is present in the nuclei and dendrites of cortical cells, the pyramidal cell layer of the Hippocampus and the Stratum radiatum/lucidum, the nuclear and molecular layer of the Cerebellum or in the nuclei and dendrites of the Caudoputamen, but not in striatopallidal fibres. In particular specific cell layers such as the pyramidal cell layer of the Hippocampus or the Purkinje cell layer of the Cerebellum display a profound clustering of Simiate (cp. Figure 4C), which is also reflected in the intense labelling of these layers in Figure 4A. These findings are consistent with our previous observations in primary hippocampal cell cultures, which also showed that Simiate is present in dendrites and nuclei and that Simiate may cluster inside the nucleus.

Again, in FMR1^-/-^ animals (Figure 4B), despite the loss in immunofluorescence intensity, no major abnormalities are seen, except for the Cerebellum (Figure 4C-D): While wildtype animals demonstrate a high degree of Simiate clustering in almost all Purkinje cells (96.26%, n=214 cells from 3 animals (N=3), Figure 4C), Purkinje cells from FMR1^-/-^ mice often lack these clusters (only 66.67% of 201 cells display a clustered distribution of Simiate in the nucleus; Figure 4D, rhombic tip arrows (n=201, N=3, Fisher's exact test p<0.001)). This result is in line with a recent report from a post-mortem study of FXS patients demonstrating several abnormalities in the Purkinje cell layer of the Cerebellum including misplaced and misoriented Purkinje cells [55].

A quantification of the protein levels in several brain regions based on immunofluorescence intensities standardized to the intensity of the Corpus callosum (Figure 4E-F) indicated further abnormalities in the Cerebellum of FMR1^-/-^ mice: Here, the Simiate signal is significantly shifted toward the molecular layer (+8.5%, t-test p<0.001) when compared to wildtype animals, suggesting an altered localisation of Simiate in this brain region. Remarkably, no comparable changes were observed in any other analysed brain region.

**Figure 5:**
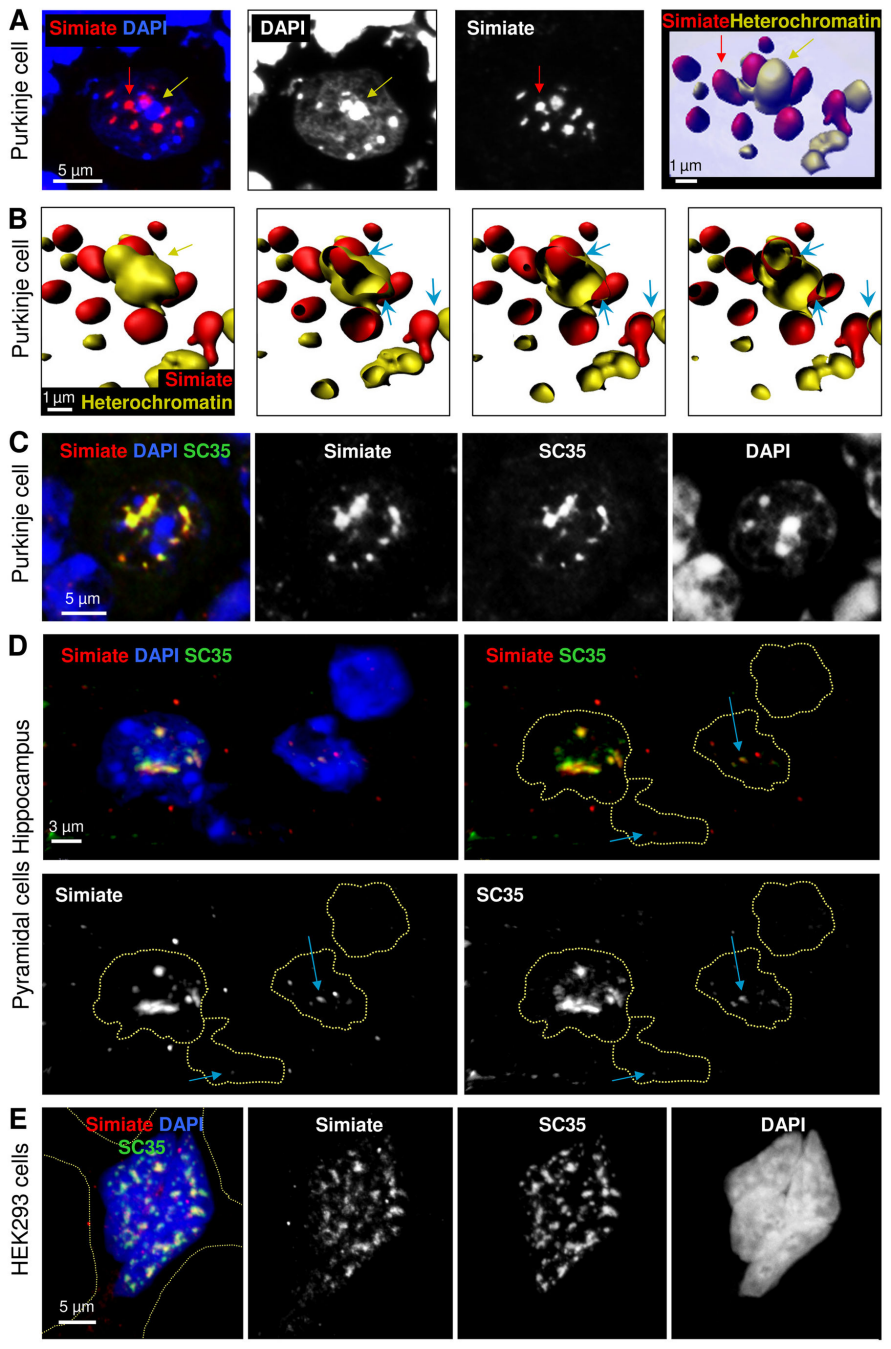
Simiate in the nucleus. A) A picture showing the nucleus of a Purkinje cell in the Cerebellum of an adult mouse brain along with a 3D reconstruction of Simiate and heterochromatin clusters. Please refer to the red and yellow arrows for orientation. B) Four virtual slices taken through the same nucleus. The blue arrows indicate areas displaying a colocalisation of Simiate and heterochromatin. C) Another Purkinje cell was treated as in A) and additionally labelled with SC35 to visualize nuclear speckles. D) Four nuclei from the CA1 pyramidal cell layer of the Hippocampus with different amounts of Simiate. The absence of Simiate coincides with the absence of nuclear speckles. Nuclear speckles of different sizes are labelled with blue arrows to illustrate the colocalisation of Simiate and SC35. For convenience, dotted circles are used to outline nuclei in all graphs not displaying DAPI. E) HEK-293 cells also express endogenous Simiate. The somata is indicated by a dotted line.

The significant alteration of Simiate clustering in cerebellar Purkinje cells from FMR1^-/-^ mice encouraged us to study these clusters in more detail. Using 3D reconstructions from z-stacks taken through nuclei, we first addressed the question of the nature of these clusters by performing co-stainings (Figure 5A-E). When DAPI (4',6-diamidino-2-phenylindole) was applied to label heterochromatin foci (Figure 5A-B) we found only little overlap (Figure 5A), although in particular the virtual slices taken through the 3D reconstruction of this nucleus (Figure 5B) revealed a partial colocalisation of Simiate clusters and heterochromatin foci. Indeed, some Simiate clusters seem to be attached to heterochromatin foci in the demonstrated manner most of the time (see arrows in Figure 5B), suggesting a functional connection. 

Aside from heterochromatin foci, nucleoli, nuclear speckles and PML nuclear bodies are other prominent compartments of the nucleus of similar appearance, however, neither nucleoli nor PML nuclear bodies match the characteristics of Simiate clusters in terms of size and shape. Hence, we utilized SC35 to stain nuclear speckles ([56]; Figure 5C-E). We found a profound colocalisation of Simiate and SC35 (Figure 5C), which is stable regardless of the amount of Simiate present inside the nucleus or the degree of clustering, respectively (Figure 5D), and independent of the cell type (Figure 5C-E) or the cell cycle phase (data not shown). Taken together, these results suggest that Simiate resides in nuclear speckles, pointing towards a function in splicing or transcription regulation events. 

**Figure 6:**
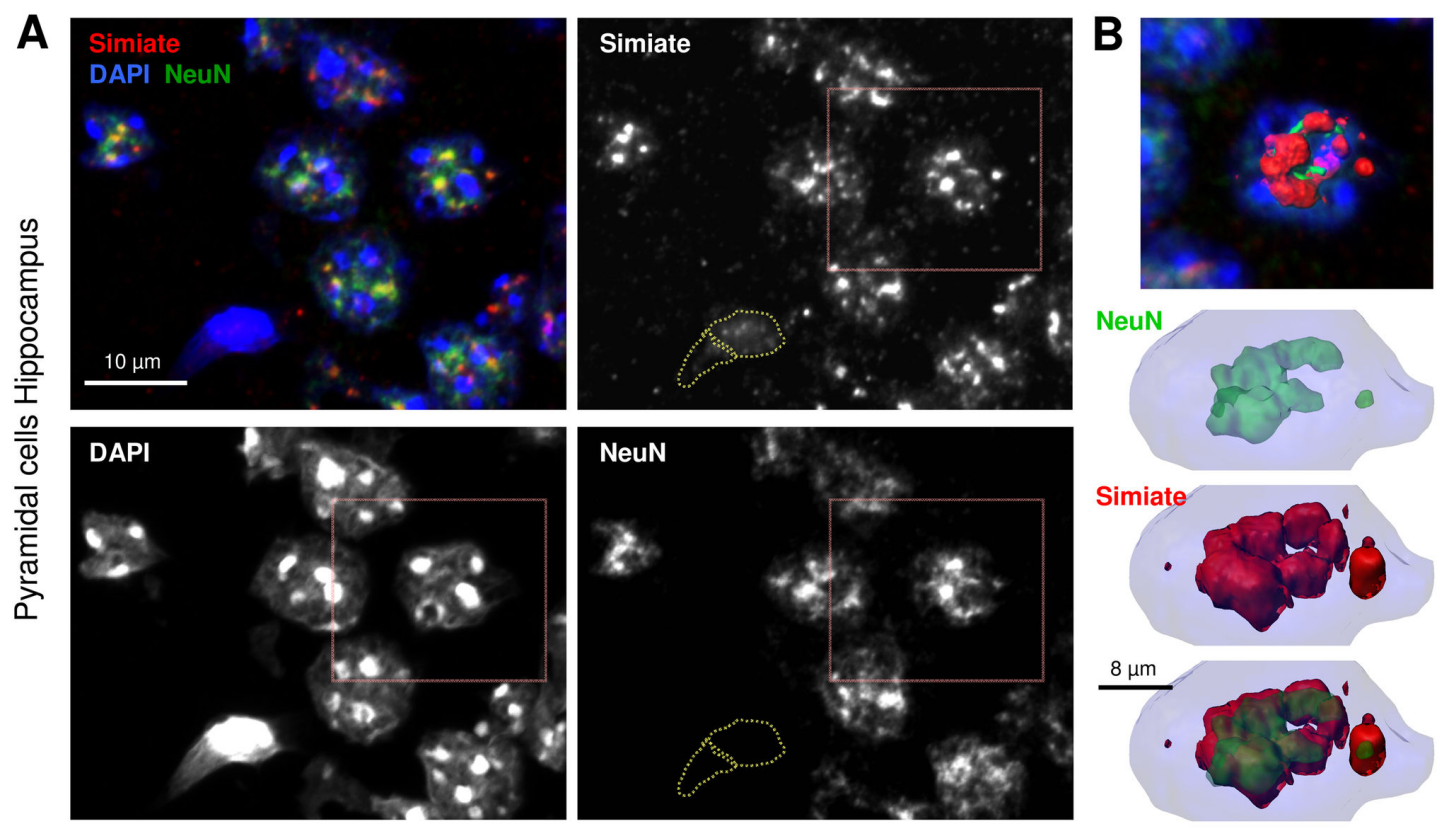
Simiate in neuronal and non-neuronal nuclei of an adult FMR1^-/-^ mouse brain. A) A part of the pyramidal cell layer of the Hippocampus. Neuronal cells are marked with NeuN. The nuclei of two glia cells located at the bottom of the picture are delineated with dotted lines in all graphs not displaying DAPI. B) 3D reconstruction of a neuronal nucleus (red box in A). NeuN is not only a marker for neuronal cells, but also known to reside in nuclear speckles.

We now set out to study eventual effects of the loss of FMRP in FXS on Simiate and nuclear speckles. Utilizing NeuN (alternative name: Fox3) to differentiate between neuronal and non-neuronal cells we confirmed the presence of Simiate in both cell types for brain slices. Interestingly, glial cells generally display a very diffuse distribution of Simiate, whereas neuronal cells show a highly clustered Simiate pattern throughout the brain (Figure 6A). The 3D reconstruction of a CA1 pyramidal cell (Figure 6B) shows that Simiate and NeuN are also colocalised in nuclear speckles, which is in accordance with recent reports on NeuN delineating that the protein is a regulator of neuronal splicing and a component of both, the nuclear matrix and nuclear speckles [57,58]. 

In order to characterize Simiate in nuclear speckles from glia and neurons in FMR1^-/-^ and wildtype animals, the total volume and total surface of all nuclear speckles of a cell were calculated for several brain regions using Simiate to outline the speckles and NeuN as reference. No significant differences between NeuN and Simiate localisation with respect to nuclear speckles were observed (Figure 6A,B).

**Figure 7:**
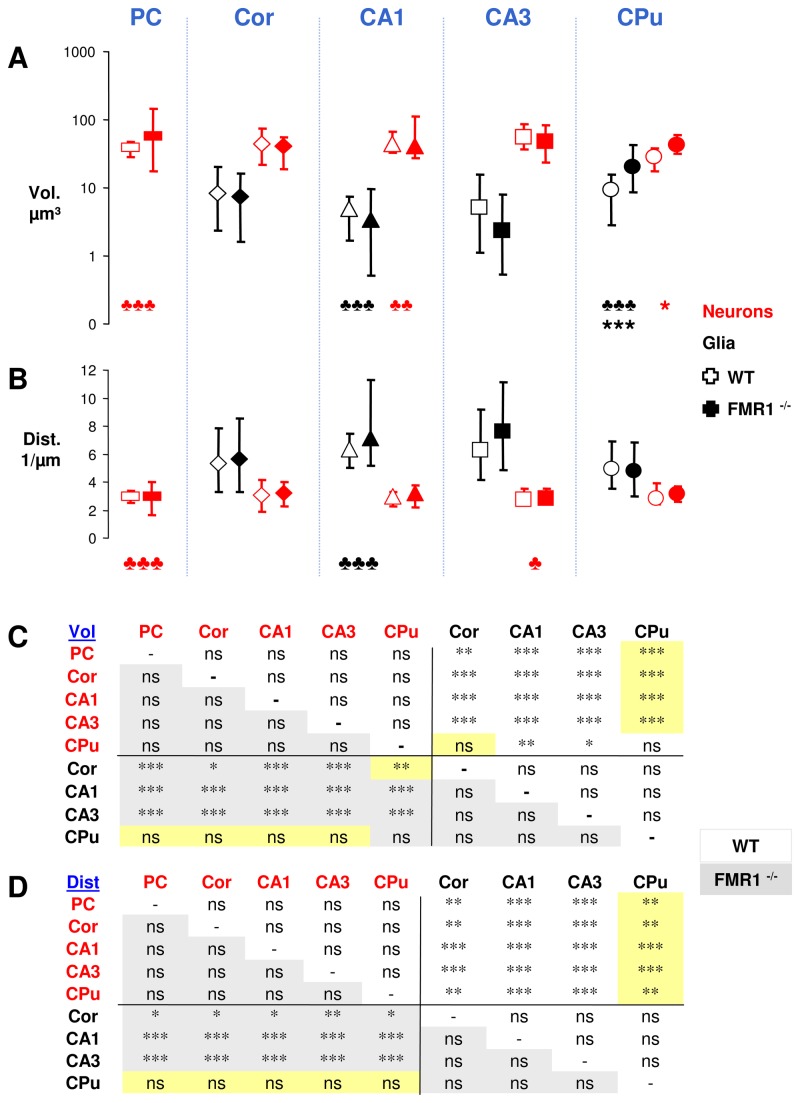
Simiate and nuclear speckles in FMR1^-/-^ mice. A,B) The graphs show the volume (Vol.; A,C) and distribution (Dist.; B,D) of nuclear Simiate in neuronal and non-neuronal cells for diverse brain regions from FMR1^-/-^ and wildtype mice. Neurons were identified by the presence of NeuN. A,B) In each column, symbols indicate the median, while the error bars display the corresponding 10/90 quantile. Stars represent significant differences between medians, clubs between variances. Each group contains 14-18 cells (n) from two independent experiments. The distribution was calculated as ratio of surface to volume. Please note the logarithmic scale in A). Results from Dunn's multiple comparison post-test of Kruskal-Wallis statistics for A) and B) are shown in C) and D), respectively. C, D) Yellow backgrounds indicate significant differences between wildtype and FMR1^-/-^. CA1,3: regions of the Hippocampus, Cor: Cortex, CPu: Caudoputamen, ns: non significant, PC: Purkinje cell, wt: wildtype.

Our results revealed that most of the differences in the manifestation of nuclear speckles are observed between glia and neurons, but not between brain regions or between wildtype and FMR1^-/-^ mice (Figure 7A-D). In general, the total volume of nuclear speckles in neurons is significantly higher than in glia (median: 40.6 vs. 5.9 µm^3^, H(8) = 110.7, p<0.001) and experiences a significant narrower range of regulation (f-test p<0.001), while neither neurons nor glia display relevant differences among brain regions. There is one exception though: neurons of the Caudoputamen demonstrate no significant deviations from cortical glia or glia of the Caudoputamen itself. Indeed, these neurons experience a slightly lower speckle volume (-19,2µm^3^ on average) than all other analysed neurons.

Aside from the notable variation between neurons and glia, which is also present in FMR1^-/-^ animals (median: 43.0 vs. 5.9 µm^3^, H(8) = 90.3, p<0.001), some more specific distinctions between FMR1^-/-^ and wildtype mice are present as well. In the Caudoputamen for instance, both, neurons and glia of FMR1^-/-^ mice experience a significant increase in the total speckle volume when compared to wildtype animals (u-test p<0.001 for neurons and p=0.016 for glia). Moreover, the variance observed in glial cells is also significantly elevated (f-test p<0.001). 

Further differences between FMR1^-/-^ and wildtype mice are found in the CA1 region of the Hippocampus: Both, glia and neurons of FMR1^-/-^ mice experience a significant increase in the volume variance (f-test p<0.001 for glia and p=0.009 for neurons). The same also applies to cerebellar Purkinje cells (f-test p<0.001), but not to cortical cells. 

Hereafter, we assessed the distribution of nuclear speckles. The relation of surface to volume served to characterise the spreading. Hence, a small number indicates a highly clustered distribution, whereas bigger numbers reflect a diffuse dispersion (Figure 7B,D). Again, most of the differences are seen between neurons and glia, but not between brain regions or between FMR1^-/-^ and wildtype animals. In wildtype mice, nuclear speckles are significantly more clustered in neurons than in glia (median: 2.9 vs. 6.0, H(8) = 95.6, p<0.001) and experience a significant narrower range of regulation (f-test p<0.001), but no relevant influence of the brain region can be detected. 

In FMR1^-/-^ mice, the difference between neurons and glia is preserved (median: 3.1 vs. 6.1, H(8)=97.1, p<0.001), except for the glial cells of the Caudoputamen, which fail to demonstrate significant distinctions from neurons. Looking at the variances, it turned out that cerebellar Purkinje cells as well as glia from the CA1 region of the Hippocampus show a significantly increased regulation range (f-test p<0.001 for the Purkinje and glial cells), whereas neurons of the CA3 region experience a significantly decreased regulation range (f-test p=0.044). No differences are seen in the Cortex.

Taken together, our data shows that in FXS, most differences in the volume and distribution of nuclear speckles are present in the Caudoputamen, a rather surprising result, since most abnormalities in FXS have been reported from the Hippocampus and Cortex thus far, however, studies of the Caudoputamen are lacking. On the other hand, the results also illustrate that an altered range of regulation in both, the volume and distribution of nuclear speckles of specific cell populations such as cerebellar Purkinje cells or glial cells of the CA1 region, characterises the effects of the disease, thereby suggesting that aside from altered translation control, aberrant splicing and/or transcription regulation are also involved in FXS.

### 3.4: Simiate and nuclear speckles

**Figure 8:**
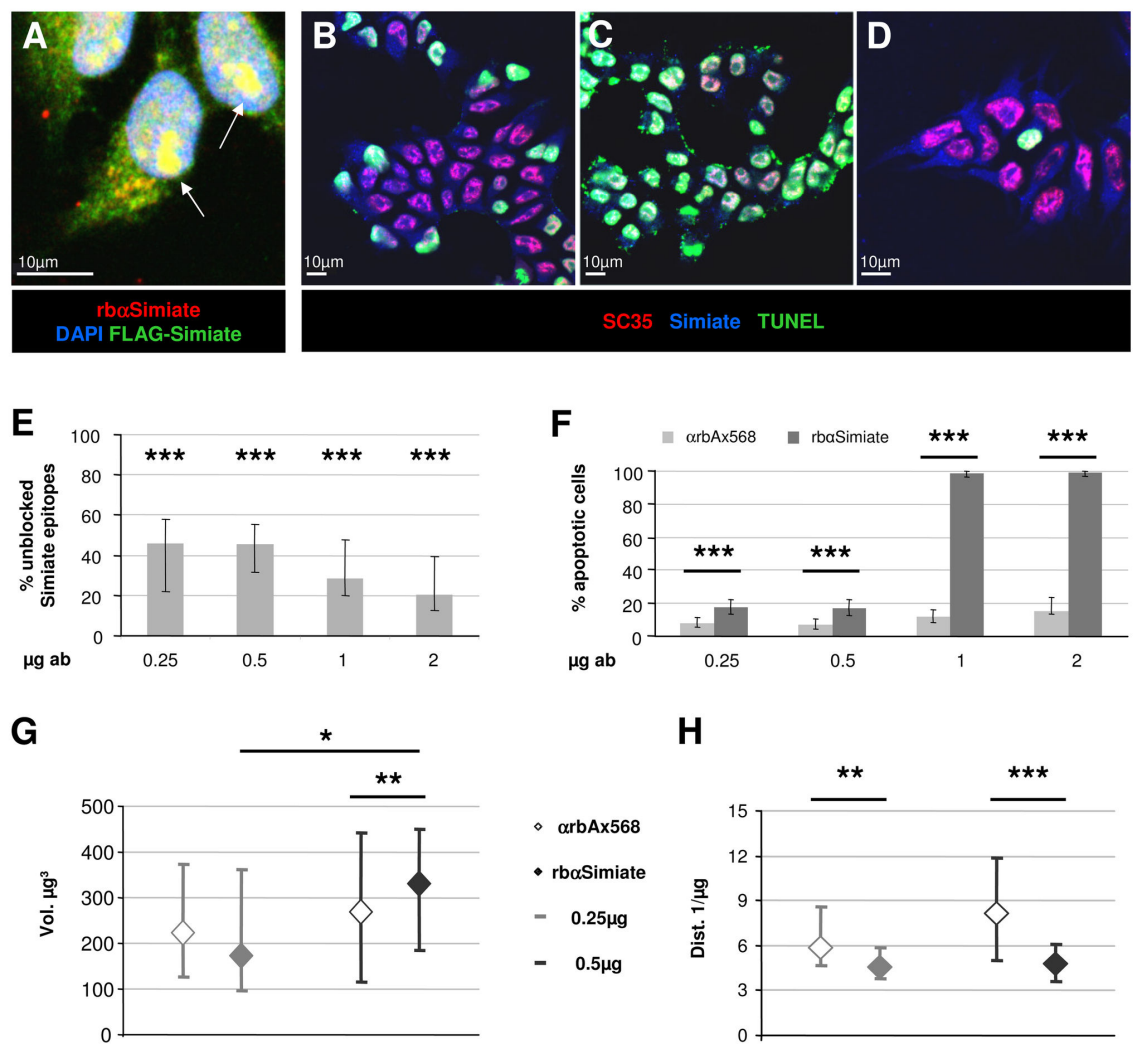
Simiate is vital to cells. A) Chariot reagent shuttled rbαSimiate (0.5µg) detects FLAG-Simiate in transfected HEK-293 cells. The nuclei are visualized by DAPI staining. The arrows indicate clusters of rbαSimiate and FLAG-Simiate immunofluorescence inside the nucleus. B-D) Apoptosis in rbαSimiate and αrbAlexa568 treated HEK-293 cells. B) 0.25µg rbαSimiate, C) 1.0µg rbαSimiate and D) 1.0µg αrbAlexa568 as negative control. TUNEL staining (in green) served to identify apoptotic cells, while nuclear speckles were outlined with SC35 (in red). E) Quantification of the amount of endogenous Simiate epitopes not targeted by antibodies. F) Quantification of apoptotic cells. The increase in the percentage of TUNEL positive cells after rbαSimiate treatment is extremely significant (Chi^2^: p<0.001) compared to the control treatment, where rbαAlexa568 was applied. n (0.25-2.0µg) rbαAlexa568: 293, 262, 276 and 276 cells and n (0.25-2.0µg) rbαSimiate: 300, 237, 241 and 252 cells. The two bottom graphs show the volume (G) and distribution (H) of nuclear speckles in rbαSimiate (filled symbols) and αrbAlexa568 (empty symbols) treated HEK-293 cells. 0.25µg antibodies are shown in light gray, whereas 0.5µg are demonstrated in dark gray. Due to massive apoptosis induced by higher amounts of rbαSimiate, those cells were not analysed. n (0.25-2.0µg) rbαAlexa568: 25, 25, 20 and 20 cells and n (0.25-0.5µg) rbαSimiate: 24 and 25 cells. Stars represent significant differences between medians.

In order to further access the role of Simiate in vivo, we decided to interfere with the function of endogenous Simiate. Small interfering RNAs (siRNA) are commonly used to silence specific genes post-transcriptionally, hence minimising endogenous expression of the corresponding protein; however, our initial experiments indicated that a reduction of Simiate expression is sufficient to induce cell death within a few hours. We therefore seeked to acutely decrease the availability of functional Simiate in a directly dose controlled manner by shuttling increasing amounts of rbαSimiate antibodies into HEK-293 cells using chariot reagent (Figure 8). To validate the assay, we expressed FLAG-Simiate in HEK-293 cells prior to the chariot treatment with rbαSimiate and analysed the colocalisation of FLAG-Simiate and rbαSimiate consecutively (Figure 8A). The experiment revealed that the antibody is able to enter the cell soma as well as the nucleus, where it colocalises with FLAG-Simiate clusters (see arrows in Figure 8A). Closely following the manufacturer's recommendations, we now used 0.25, 0.5, 1.0 and 2.0µg of rbαSimiate to block the endogenous protein epitopes (representative in Figure 8B-C), while a corresponding amount of αrbAlexa568 served as negative control (Figure 8D). Importantly, the treatment with αrbAlexa568 had no effect on the welfare of the cells, independent from the concentration used (Figure 8D, E), foreclosing a toxic impact of the treatment. The application of rbαSimiate had no evident effect on the localisation pattern of endogenous Simiate, in particular the colocalisation with SC35 and the fine punctured signal throughout the soma remained preserved. A quantification of the amount of unblocked Simiate epitopes revealed that we were able to target up to 80% of the rbαSimiate binding sites, using 2.0µg of antibodies (Figure 8E). 

Further analyses established that amounts of rbαSimiate ranging from 0.25 to 0.5µg were sufficient to induce significantly elevated rates of cell death within 3 hours after treatment as measured by TUNEL staining (0.25µg: 17.3% vs. 7.9% control; 0.5µg: 16.9% vs. 6.9% control; both Fisher’s test p<0.001; n=237-300), whereas amounts of 1.0µg or more led to apoptosis in almost all cells of the culture (99.2% vs. 15.2% control; Fisher’s test p<0.001; n=241-276; Figure 8B-D,F). This result is also reflected in an increased number of cells dismounted from the cover slips and present in the medium (data not shown). By contrast, elevated amounts of Simiate, as achieved by the additional expression of FLAG-Simiate in HEK-293 cells (Figure S4), had no effect on cell survival, suggesting that Simiate is also in increased quantums not toxic to cells, but necessary.

To address the effects of Simiate blockage in more detail, we performed 3D reconstructions of nuclear speckles in non-apoptotic HEK-293 cells treated with either 0.25 and 0.5µg rbαSimiate or 0.25-2.0µg αrbAlexa568 using SC35 to outline the speckles (Figure 8G-H). While no significant differences in the volume, area and distribution of nuclear speckles are observed within the control group, a highly significant increase in the volume of nuclear speckles is seen when comparing 0.5µg control and rbαSimiate treated cells (medians: 38.8 and 76.0; H(5)=16.84, p=0.005, posthoc test p<0.01). Also, 0.5µg rbαSimiate treated cells display a significant higher speckle volume than 0.25µg rbαSimiate treated HEK-293 cells (medians: 39.5 vs. 76.0; p<0.05; Figure 8G). Looking at the surface area of nuclear speckles, no major differences are noticed except for a significant elevated level present in 0.5µg rbαSimiate treated cells when compared to 0.25µg rbαSimiate treated cells (medians: 173.8 vs. 332.5; H(5)=0.006; posthoc test p<0.01). By contrast, the analysis of the speckle distribution revealed a highly or, respectively, extremely significant decrease in the speckle spreading of 0.25 and 0.5µg rbαSimiate treated HEK-293 cells when compared to the corresponding control cells (medians: 5.8 vs. 4.6 and 8.1 vs. 4.7; H(5)=39.92, p<0.001, posthoc tests p<0.01 and 0.001; Figure 8H). These findings show that the loss of functional Simiate leads to an agglomeration of nuclear speckles and quickly induces apoptosis.

## Discussion

Here, we present a novel protein named Simiate, which links FMRP to transcription and splicing control in nuclear speckles. Although FMRP has been found to be involved in many aspects of the mRNA metabolism and even been indicated to function in chromatin remodelling and alternative splicing of its own transcript via an association with G-quadruplexes located in the coding region [22-25], nuclear speckles have not been implicated in FXS yet. Using different molecular, biochemical and immunocytochemical methods, we show that Simiate is an evolutionary old protein present in all analysed species from protists to mammals and whose mRNA is recognized by FMRP. Accordingly, FMR1^-/-^ mice, a model for FXS, display brain-region specific changes in the expression and/or localisation of Simiate along with alterations in the appearance of nuclear speckles, suggesting that the interaction of FMRP with the Simiate transcript is not only relevant to the function of Simiate, but also highly regulated.

### 4.1: Simiate and FMRP

Performing *in silico* analyses of the mRNA of Simiate we found that the transcript contains several motifs which may mediate the demonstrated association with FMRP: 8 U-pentamers and 11 G-quadruplexes, of which 3 are composed of ACUK/WGGA motifs as identified by Ascano et al. [17] in app. 50% of their transcripts. While there is only little known about the interaction of U-pentamers and FMRP (reviewed in 49), G-quadruplexes have been studied in great detail since they were discovered as RNA motifs recognized by FMRP ([46-48]; reviewed in 22). These investigations revealed that G-quadruplexes in the 5' UTR are likely to mediate a translational repression, whereas G-quadruplexes in the 3' UTR may act as a translational activator though their function is less clear since in some cases, such as PSD95, conflicting results were obtained. Indeed, RNA motifs located in the 3' UTR have mostly been associated with transport control, but since efficient mRNA transport is a prerequisite for effective translation, it is likely that further and multiple mechanisms regulating the protein expression via 3' UTR motifs are in effect. However, these possibilities have not been explored for G-quadruplexes yet. Given that most of the U-pentamers present in the Simiate transcript -7 out of 8- are localised in the 3' UTR, it is therefore tempting to speculate that FMRP could control the expression of Simiate in two ways: via U-pentamer mediated transport regulation and/or via G-quadruplex conveyed translation control.

This idea is in line with our observations of FMR1^-/-^ animals. Despite an overall decrease in protein levels, a Cerebellum specific shift of the Simiate signal towards the molecular layer is observed, implying a mislocalisation of Simiate possibly toward the dendritic tree of Purkinje cells and/or eventually toward the protrusions of Neuroglia cells. This condition clearly requires highly specific regulation mechanisms, hence favouring several different and differentially localised interaction options over a single binding motif. On the other hand, the result also rises the question why we did not observe any further changes in the expression of Simiate, for example in the pyramidal cell layer or the Stratum radiatum. Given that the observed signal shift is rather small (8.5%) and that further brain-region specific differences between wildtype and FMR1^-/-^ animals were found in nuclear speckles using 3D reconstructions, it is likely that the method was not sensitive enough to detect more (subtle) changes in the localisation of Simiate, which may nonetheless be present, but most likely only concern certain cell populations, making it hence difficult to detect these changes in brain slices.

### 4.2: Simiate and transcription

Our results have shown that inside the nucleus, Simiate strictly resides in speckles as outlined with two different antibodies specific to the nuclear speckle proteins SC35 and NeuN, still they also illustrated that Simiate and nuclear speckles frequently affix to heterochromatin foci, implying a functional connection. Indeed, this observation is in accordance with another study [27] demonstrating that nuclear speckles can be involved in the generation of an euchromatin environment in the neighbourhood of heterochromatin resulting in an attachment of heterochromatin foci and euchromatin to nuclear speckles. 

Interestingly, the size, shape and number of nuclear speckles have been associated with alterations in the transcription rate. While the inhibition of transcription has been connected with an increase in speckle size, enhanced transcription rates have been linked to decreased speckle sizes and increased numbers, reflecting a shift from a highly clustered appearance to a diffuse spreading of speckles in the nucleus after gene activation [30-34]. These investigations evince that the alterations in nuclear speckle volume and distribution we found represent altered rates of gene transcription and/or RNA splicing in specific cell populations of FMR1^-/-^ mice. Though it is plausible that FMRP impinges on gene transcription and RNA splicing in one way or the other, current research has focused on the role of FMRP in translation regulation and the control of FMR1 transcription, whereas this aspect of protein expression modulation has not been studied yet.

Most of the differences we observed in nuclear speckles are found between neurons and glia, with glial cells displaying a nuclear speckle pattern characteristic for higher levels of gene transcription and RNA splicing than neurons. This finding is consistent with the extensive role of glial cells in maintaining homeostasis and supplying nutrients to ensure correct neuronal function. However, some specific alterations between wildtype and FMR1^-/-^ mice are seen as well: In particular the variance in the volume and distribution of nuclear speckles is enhanced in different brain regions of FMR1^-/-^ animals, including the CA1 region of the Hippocampus or the Purkinje cell layer of the Cerebellum. Both brain regions have been implicated in FXS ([55], reviewed in 59). Intriguingly though, most differences in the volume and distribution of nuclear speckles are discovered in the Caudoputamen, a brain region which to date has not been connected with FXS, although recent research has shown that the Caudoputamen is involved in reinforcement and implicit learning and in some cases even interacts with the Hippocampus (reviewed in 60). Since the observed increase in the variance in gene transcription and/or RNA splicing among cells from FMR1^-/-^ mice reflects an extended range of regulation to both extremes, these results imply that a lack of control on two basic molecular mechanisms highly relevant to learning and memory is involved in the mental retardation phenotype characteristic for FXS. This idea is further substantiated by recent research demonstrating that alterations in the morphology of nuclear speckles occur in several diseases associated with intellectual deficits including the FRAXE mental retardation [56,61], which -though less severe- closely resembles the phenotype observed in FXS, as well as Rett- and Down syndrome [62-64]. All these diseases share changes in the organisation of nuclear speckles and functional alterations in transcription and/or splicing pathways leading to misregulated protein expression.

Remarkably, the blockage of endogenous Simiate epitopes with antibodies specific to Simiate in HEK-293 cells revealed that Simiate is fundamental for cell survival. This result is in line with the presence of Simiate in primary hippocampal cultures from div 1 on, the ubiquitous expression of the protein among various types of tissue and the development of Simiate early in evolution when eukaryotes first occurred. Furthermore, the agglomeration of nuclear speckles induced by the blockage of Simiate suggests that Simiate acts as a transcription and/or splicing enhancer of – considering the rapid cell death induced by the blockage – either the corresponding machinery itself or certain indispensable genes. These findings further support the idea that the alterations observed in nuclear speckles of FMR1^-/-^ mice and hence, in transcription and/or splicing rates, are indeed related to malfunctioning Simiate.

Taken together, our data suggests a model where FMRP controls the expression of Simiate and Simiate in turn controls gene transcription and/or RNA splicing in nuclear speckles and thereby contributes to protein expression regulation.

## Supporting Information

Figure S1 **Simiate orthologs.** Alignment of Simiate orthologs from manifold species. The amino acids are represented as follows: Blue: hydrophobic amino acids (V,L,I,P,L,M), red: acidic amino acids (D,E), green: amino acids (H,K,R), purple: aromatic amino acids (F,Y,W), yellow: small amino acids (G,A), black: nucleophilic amino acids (S,T,C). Since bacteria and archaea express GCSHPP (glycine cleavage system H protein, mitochondrial precursor) only, both groups are shown in italic letters. The black box indicates a region containing conserved amino acids.
(TIF)

Figure S2 **Characterisation of Simiate specific antibodies.** A-E, A'-E') Simiate is specifically detected by the newly generated rabbit- as well as guinea pig- anti-Simiate antibody (rb/gpαSimiate). A, A') Immunofluorescence images illustrating the colocalisation of GFP-Simiate (green) and rb/gpαSimiate signal (red) in HEK-293 cells. The nuclei are marked with DAPI (blue). B, B') Preincubation of rb- and gpαSimiate with GST-Simiate blocks the signal completely in both cases. C, C') Rb and gpαSimiate recognize Simiate in western blots. HEK-293 cells were transfected with either GFP-Simiate or GFP, lysed and subjected to SDS-PAGE, whereupon blots were stained with either αGFP, pre-immune serum (Pre-IS; 1:500), rb- and gpαSimiate, or with GST-Simiate preincubated rb/gpαSimiate. Protein amounts per lane: GFP 80ng and GFP-Simiate 40ng. D, D') Mouse brain cytosol. Numbers indicate the molecular weight in kDa. 400µg protein in total. The upper part (40-150 kDa) in D’ represents a 444s exposure, while the lower part (10-25 kDa) shows a 300s exposure, illustrating that even on an extended exposure no additional proteins are detected. E, E') The detection limit of rb- and gpαSimiate amounts to app. 20ng GFP-Simiate in western blots. Negative control: 80ng GFP in HEK-293 cell lysate.
(TIF)

Figure S3 **Simiate expression is reduced in the FMR1^-/-^ mouse brain.** Mouse brain from either 6 FMR1^-/-^ or WT mice were homogenized (MBH) and samples were subjected to SDS-PAGE (400µg protein in total). The following western blot was stained anti-Calnexin to rate available amounts of protein and anti-Simiate to evaluate the different expression levels of Simiate in FMR1^-/-^ or WT mice (cp. Immunofluorescence in Figure 4). After an exposure time (Exp.) of 200s, Simiate can be detect in the WT, but not in the FMR1^-/-^ brain homogenate, however, after an exposure time of 600s, Simiate also appears in the FMR1^-/-^ sample. 
(TIF)

Figure S4 **Enhanced expression of Simiate does not induce apoptosis.** Representative immunofluorescence stainings of HEK-293 cells, which expressed a FLAG-Simiate construct for 24h (A) and 48h (B) or FLAG alone for 48h (C). TUNEL staining (in green) served to identify apoptotic cells, while the nuclei were outlined with DAPI (in blue). D) Quantification of apoptotic cells (%) after 24h or 48h of FLAG-Simiate expression: The increased expression of Simiate has no discernible effect of the amount of TUNEL positive cells compared to FLAG transfected cells (Chi²: p0.3;ns). n (24h,48h) FLAG-Simiate = 213, 204 cells and n (24h,48h) FLAG = 201, 204 cells.
(TIF)
