# Supplementary figures and images for: Identification and Characterisation of Simiate, a Novel Protein Linked to the Fragile X Syndrome
Source: PLoS One. 2013 Dec 11;8(12):e83007. doi: 10.1371/journal.pone.0083007 (PMC3859600; doi:10.1371/journal.pone.0083007)

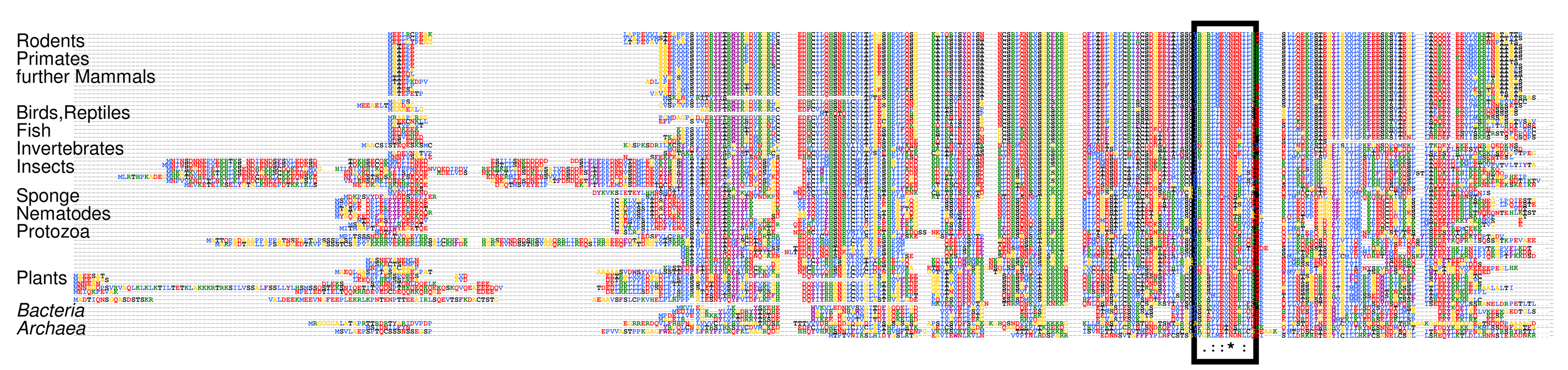

Supplement: Figure S1 — Simiate orthologs. Alignment of Simiate orthologs from manifold species. The amino acids are represented as follows: Blue: hydrophobic amino acids (V,L,I,P,L,M), red: acidic amino acids (D,E), green: amino acids (H,K,R), purple: aromatic amino acids (F,Y,W), yellow: small amino acids (G,A), black: nucleophilic amino acids (S,T,C). Since bacteria and archaea express GCSHPP (glycine cleavage system H protein, mitochondrial precursor) only, both groups are shown in italic letters. The black box indicates a region containing conserved amino acids. (TIF) [file pone.0083007.s001.tif]

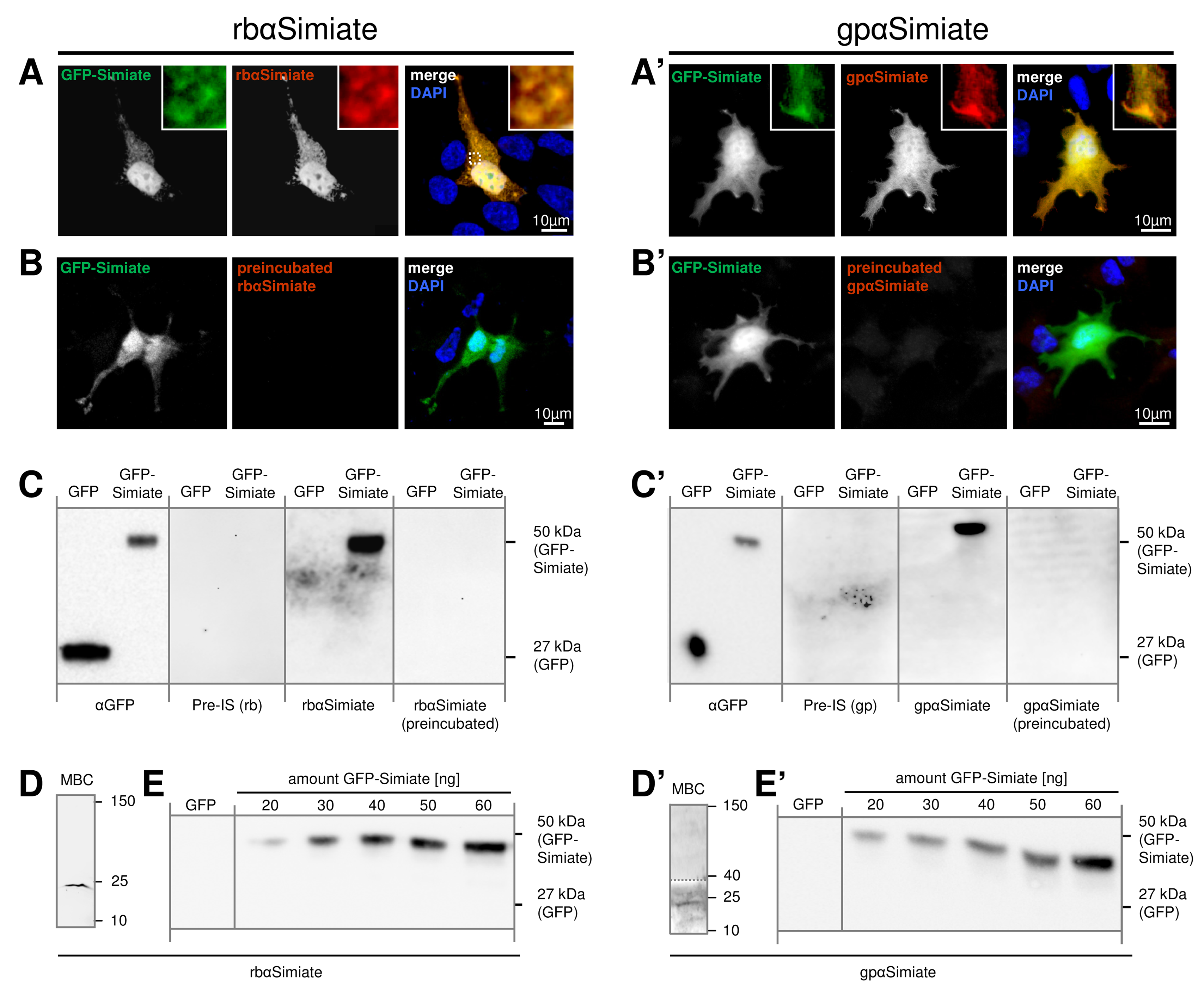

Supplement: Figure S2 — Characterisation of Simiate specific antibodies. A-E, A'-E') Simiate is specifically detected by the newly generated rabbit- as well as guinea pig- anti-Simiate antibody (rb/gpαSimiate). A, A') Immunofluorescence images illustrating the colocalisation of GFP-Simiate (green) and rb/gpαSimiate signal (red) in HEK-293 cells. The nuclei are marked with DAPI (blue). B, B') Preincubation of rb- and gpαSimiate with GST-Simiate blocks the signal completely in both cases. C, C') Rb and gpαSimiate recognize Simiate in western blots. HEK-293 cells were transfected with either GFP-Simiate or GFP, lysed and subjected to SDS-PAGE, whereupon blots were stained with either αGFP, pre-immune serum (Pre-IS; 1:500), rb- and gpαSimiate, or with GST-Simiate preincubated rb/gpαSimiate. Protein amounts per lane: GFP 80ng and GFP-Simiate 40ng. D, D') Mouse brain cytosol. Numbers indicate the molecular weight in kDa. 400µg protein in total. The upper part (40-150 kDa) in D’ represents a 444s exposure, while the lower part (10-25 kDa) shows a 300s exposure, illustrating that even on an extended exposure no additional proteins are detected. E, E') The detection limit of rb- and gpαSimiate amounts to app. 20ng GFP-Simiate in western blots. Negative control: 80ng GFP in HEK-293 cell lysate. (TIF) [file pone.0083007.s002.tif]

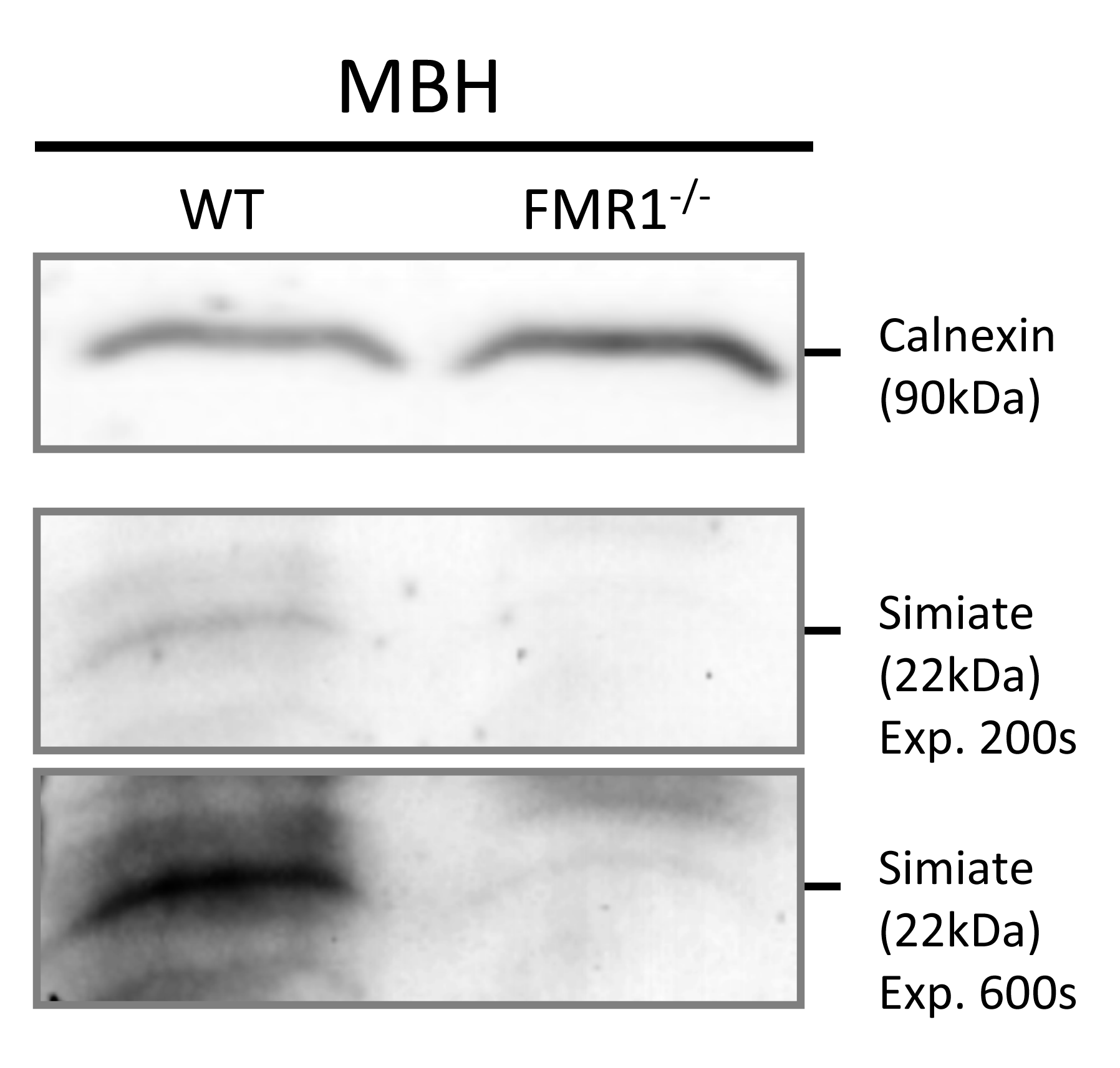

Supplement: Figure S3 — Simiate expression is reduced in the FMR1-/- mouse brain. Mouse brain from either 6 FMR1-/- or WT mice were homogenized (MBH) and samples were subjected to SDS-PAGE (400µg protein in total). The following western blot was stained anti-Calnexin to rate available amounts of protein and anti-Simiate to evaluate the different expression levels of Simiate in FMR1-/- or WT mice (cp. Immunofluorescence in Figure 4). After an exposure time (Exp.) of 200s, Simiate can be detect in the WT, but not in the FMR1-/- brain homogenate, however, after an exposure time of 600s, Simiate also appears in the FMR1-/- sample. (TIF) [file pone.0083007.s003.tif]

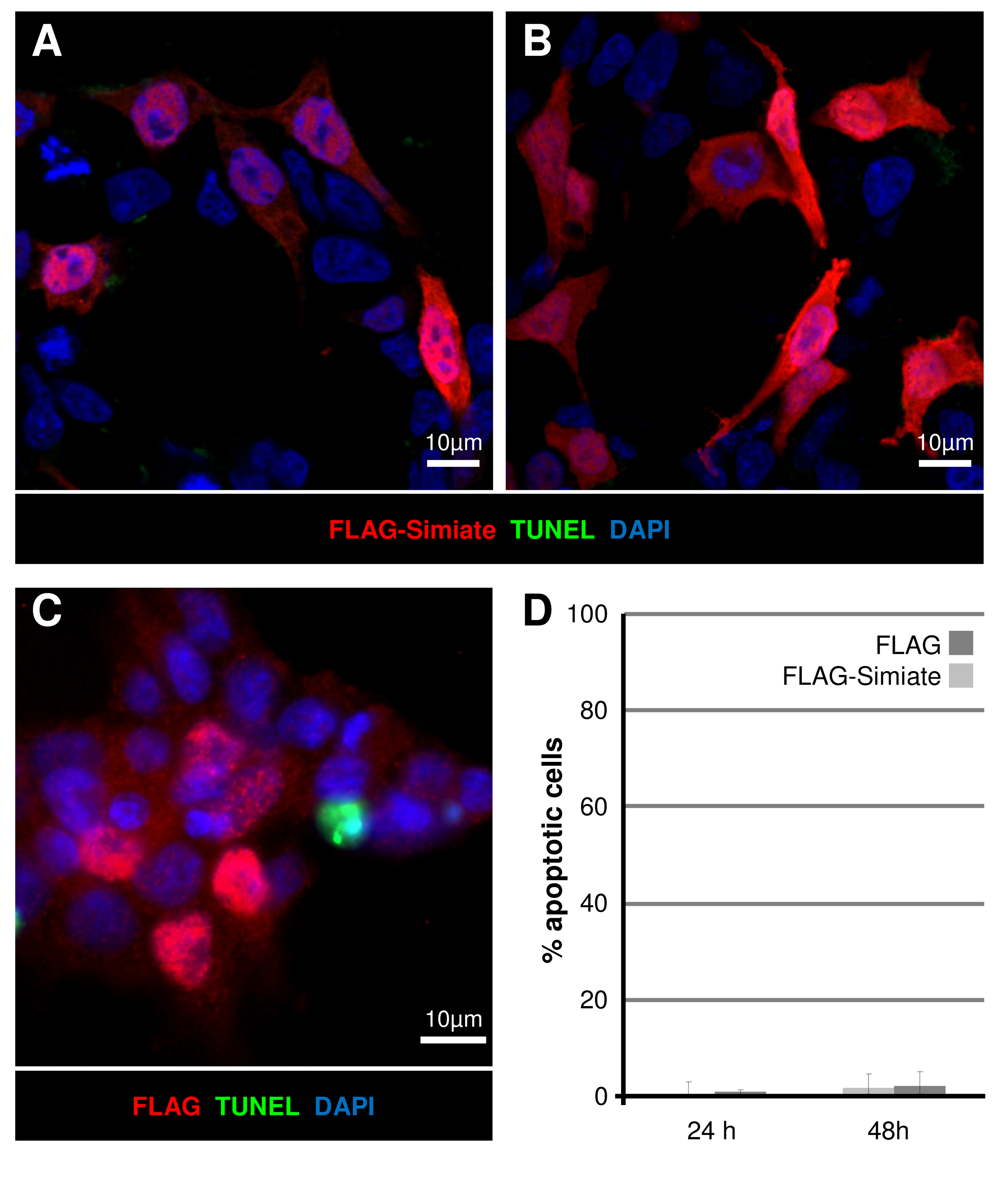

Supplement: Figure S4 — Enhanced expression of Simiate does not induce apoptosis. Representative immunofluorescence stainings of HEK-293 cells, which expressed a FLAG-Simiate construct for 24h (A) and 48h (B) or FLAG alone for 48h (C). TUNEL staining (in green) served to identify apoptotic cells, while the nuclei were outlined with DAPI (in blue). D) Quantification of apoptotic cells (%) after 24h or 48h of FLAG-Simiate expression: The increased expression of Simiate has no discernible effect of the amount of TUNEL positive cells compared to FLAG transfected cells (Chi²: p0.3;ns). n (24h,48h) FLAG-Simiate = 213, 204 cells and n (24h,48h) FLAG = 201, 204 cells. (TIF) [file pone.0083007.s004.tif]
